# Quick identification of acute chest pain patients study (QICS)

**DOI:** 10.1186/1471-2261-9-24

**Published:** 2009-06-15

**Authors:** Hendrik M Willemsen, Gonda de Jong, René A Tio, Wybe Nieuwland, Ido P Kema, Iwan CC van der Horst, Mattijs Oudkerk, Felix Zijlstra

**Affiliations:** 1 Department of Cardiology, University Medical Center, Groningen, The Netherlands; 2 Department of Radiology, University Medical Center, Groningen, The Netherlands; 3 Department of Laboratory Medicine, University Medical Center, Groningen, The Netherlands

## Abstract

**Background:**

Patients with acute chest pain are often referred to the emergency ward and extensively investigated. Investigations are costly and could induce unnecessary complications, especially with invasive diagnostics. Nevertheless, chest pain patients have high mortalities. Fast identification of high-risk patients is crucial. Therefore several strategies have been developed including specific symptoms, signs, laboratory measurements, and imaging.

**Methods/Design:**

The Quick Identification of acute Chest pain Study (QICS) will investigate whether a combined use of specific symptoms and signs, electrocardiography, routine and new laboratory measures, adjunctive imaging including electron beam (EBT) computed tomography (CT) and contrast multislice CT (MSCT) will have a high diagnostic yield for patients with acute chest pain. All patients will be investigated according a standardized protocol in the Emergency Department. Serum and plasma will be frozen for future analysis for a wide range of biomarkers at a later time point. The primary endpoint is the safe recognition of low-risk chest pain patients directly at presentation. Secondary endpoint is the identification of a wide range of sensitive predictive clinical markers, chemical biomarkers and radiological markers in acute chest pain patients. Chemical biomarkers will be compared to quantitative CT measurements of coronary atherosclerosis as a surrogate endpoint. Chemical biomarkers will also be compared in head to head comparison and for their additional value.

**Discussion:**

This will be a very extensive investigation of a wide range of risk predictors in acute chest pain patients. New reliable fast and cheap diagnostic algorithm resulting from the test results might improve chest pain patients' prognosis, and reduce unnecessary costs and diagnostic complications.

## Background

Frequently new onset chest pain is a sign of myocardial ischemia or even (threatening) myocardial infarction. Despite continuous improvements in diagnostic tools it can still be difficult to differentiate between a benign and a potential hazardous nature of acute chest pain. Percentages as high as 5% of chest pain patients presenting in the emergency room suffer or will suffer from myocardial infarction without being recognized as having an acute coronary syndrome (ACS) at presentation [1]. The consequences can be disastrous, especially when these patients would not be admitted for rhythm surveillance. A component of failure to recognize threatening myocardial ischemia seems to be that discriminating non typical from typical chest pain does not differentiate well enough between threatening myocardial ischemia or not [1,2]. Several studies have evaluated the diagnostic accuracy of a standard diagnostic flow chart to identify patients with myocardial ischemia and showed good results. Therefore, diagnostic testing at presentation or during initial hospital admittance seems warranted. Shoyeb and colleagues confirmed that patients who had diagnostic tests during initial hospital admittance had better prognosis [3]. This is why a quick but diagnostic evaluation of new onset acute chest pain patients is needed.

### New radiological techniques

One of the introduced diagnostic techniques developed is electron beam computed tomography (EBT) scan investigating the presence of coronary calcium. EBT scanning and evaluating takes less than 20 minutes [4], and has a low radiation load [5]. The technique doesn't require contrast. In review and expert opinion [4], a negative test on coronary calcium is considered to make the presence of vulnerable plaques or obstructive coronary artery disease very unlikely. A positive test quantifies coronary atherosclerosis. It seems very clear that EBT investigations negative for calcium detection, practically rule out any risk ofa coronary event in the next years [4]. On the other hand, high scores are related to a high cardiac risk, and occlusive coronary disease [6]. Although calcium scores do not have an additional risk stratifying value in high risk patients [7], it can have a great discriminating value in intermediate risk patients [8]. One of the recent large EBT-calcium score studies, the St. Francis Heart Study [9], strikingly identified high risk patients (relative risk 9.2). It was identified in patients with a calcium score >100 (a volume score). With this it was 5 times more discriminative than CRP.

Another type of CT scanning in coronary artery disease (CAD) has evolved over the last decade. Multi slice CT (MSCT) using contrast has made a true revolution in non invasive coronary imaging, resulting in near optimal coronary stenosis detection in recent studies in experienced hands and under optimal conditions [10]. 16-slice Multi detector CT (MDCT) coronary angiography was already promising for detection of significant coronary artery disease in hospital admitted patients [11]. With the use of 64-slice MDCT in to the hospital admitted patients the accuracy for detecting and ruling out significant coronary artery disease improved further, and non calcified plaques could be evaluated as well [12]. Modern 64-slice MDCT has an improved resolution. Spatial resolution has improved from 1.0 × 1.0 × 0.75 millimetres voxels to 0.4 × 0.4 × 0.4 millimetres voxels at an improved temporal resolution of 188 milliseconds. This results in such a detailed and non motion artefact sub millimetre visualisation of the coronary arteries that 72% of the CT coronary angiography has exact the same result as a conventional coronary angiography. Furthermore, amount of non-assessable segments is seriously reduced [13]. Although probably rare, a recent study using a 64-slice MDCT showed negative calcium scores in patients who did have ACS [14]. CTA with a 64-slice MDCT might be a better investigation for ruling out ACS [15]. It is also more cost effective than standard care as usual for low risk chest pain patients with nuclear testing.

Accurate detection of (significant) coronary stenoses could predict short-term cardiac risk. Correct identification of the nature of atherosclerotic plaques, especially cholesterol rich, dissected or thrombotic plaques could be direct proof of ACS in chest pain patients[16]. Because of these favourable developments in EBT and MSCT investigations, we think that EBT and MSCT are promising for future use in evaluation of CAD in outpatient stable chest pain patients. Surprisingly, EBT (and MSCT) have rarely been evaluated for analysis of new onset acute chest pain patients presenting at the emergency department. To our knowledge EBT has been evaluated limited and only for short term outcome in the emergency department [17,18]. Furthermore, in these studies calcium score was not always performed directly at presentation. Longer follow up is only evaluated in one other study with a series of 192 patients presenting with chest pain in the emergency department [19]. A negative calcium score on EBT ruled out the chance of cardiac events during 50 months follow up, and the calcium score lowered the risk ratios of traditional risk factors. 64-slice MDCT is merely tested for new onset acute chest pain patients presenting at the emergency ward [12,14]. 64-slice MDCT ruled out ACS when no plaques or stenosis could be detected. This further indicates that CT evaluation could be discriminating between acute chest pain patients with moderate to good prognosis, and patients with threatening or present cardiac events. Small studies in acute chest pain patients proved this [12,14]. MSCT angiography has not been tested on a large scale and is potentially harmful because radiation and contrast exposure. Therefore, we will execute a stepwise approach. In patients with intermediate risk first a coronary calcium score with EBT will be performed. In case of an Agatston score > 10, a MSCT coronary angiography will be performed. Unfortunately EBT technology has gradually been replaced by MSCT technology, which despite a high correlation still shows considerable variation especially in the lower range [20]. In our flowchart, in case of non cardiac cause of chest pain, MSCT will only be performed to rule out aortic dissection and pulmonary embolism a clinical suspicion. This will not be a main objective of our protocol. MSCT has already been shown successful to triple rule out all of the potentially life threatening main chest pain causes, namely aortic dissection, pulmonary artery embolism and acute coronary syndrome in small studies [21,22]. In addition calcium scoring may overlook unstable plaques without calcification. In our protocol the patients' presentation therefore strongly guided the direction within the flow-chart. In patients with a low probability, CT angiography will be performed to rule out significant CAD only in case of a high calcium score.

### Biochemical markers

There is accumulating evidence that basal inflammatory activity represents atherosclerotic disease [23]. Therefore biomarkers reflecting inflammatory activity might represent the amount of systemic and the amount of coronary atherosclerosis at the same time. It has been suggested that inflammatory biomarkers are related to the amount of systemic and coronary atherosclerosis. We will further investigate the relationship with the calcium score. On the other hand biomarkers might have additional value on top of the calcium score. Biomarkers may reflect the unstable nature of atherosclerosis, or may induce instability itself. Adhesion molecules are necessary for plaque infiltration by inflammatory cells. Oxidative stress is an important intraplaque stimulator of inflammation. Ruptured plaques cause platelet adhesion and activation which may therefore add to the diagnostic strategy in ACS.

Myocardial ischemia is the result of ACS. A problem might be that the ECG in the acute stadium and stress testing in a later stadium may not be diagnostic. Because ischemia should be detected as early as possible because of possible lethal complications, ischemic biomarkers could be very important for diagnostic purposes. Testing of biomarkers becomes more easy, cheaper and faster, and multimarker testing in ACS has already proven prognostic value [24]. On the other hand it has to be established for each individual biomarker that is extensively expressed in the coronary unstable plaque during ACS, whether its release into the systemic circulation is high enough for detection.

Table 1 shows biomarkers that have been tested ACS, in short their mechanism of action in coronary syndromes, and their observed significance in prognosis. The ideal marker or combination of markers should safely rule out ACS early after onset of chest pain.

**Table 1:** shows an overview of different biomarkers that have been tested in ACS, organized by major pathophysiologic mechanism.

**Main mechanism**	**Biomarker (ref)**	**Population tested**	**Risk prediction**
Inflammation	CRP [27,28]	asymptomatic subjects n = 1492 ACS population of Frisc trial	RR = 1.79 for CRP > 3.0 mg/L RR = 3.40 for CRP > 10.0 mg/L
	IL6 [29,30]	ACS patients n = 43 ACS patients of Frisc II trial n = 3269	OR = 7 for in-hosptial events RR = 3.47 (12 month mortality) for the non-invasive group RR = 1.43 for the invasive group Asymptomatic:RR 1.31 ACS > RR 3.5
	IL1 [29]	ACS patients n = 43	OR = 11 for in hospital events
	MCP1 [31]	ACS n = 2270 (OPUS-TIMI16) control n = 227	HR = 1.53 for death or non-fatal MI after 10 months
	IL18 [32]	n = 1229 stable/instable patients CAG reffered	HR = 3.3 for cardiovascular death in the highest vs. the lowest quartile
	IL 10 [33]	ACS n = 547 CAPTURE study	HR = 0.38 for death or non-fatal MI after 6 months
	SAA [34]	ACS n = 435	RR = 9.7 for 14 day mortality (quintile 5 vs quintiles 1–4)
	CRP [24,28]	ACS n = 965 ACS n = 985 FRISC trial	RR = 1.35 for death or non fatal MI after 6 months RR = 2.6 for cardiac death after 2 years
	Fibrinogen [24]	ACS n = 985 FRISC trial	RR = 1.99 for cardiac death after 2 years
	ICAM [35-38]	elective coronary angiography n = 1246 ACS n = 91 vs control n = 24 ACS n = 114	higher in cardiovascular death, not an independent predictor
	VCAM [35-38]	elective coronary angiography n = 1246 ACS n = 91, vs control n = 24 ACS n = 114	RR = 2.8 for cardiovascular events after 2.7 years Higher levels up till 6 months after ACS. Levels higher in patients with MACE OR = 4.3 for MACE (highest vs. lowest quartile)
	sE-selectine [35-38]	elective coronary angiography n = 1246 ACS n = 91 vs control n = 24 ACS n = 114	higher in cardiovascular death, not an independent predictor
	PlGF [39]	UAP n = 544	OR = 3.3 for death median FU 4 years
Oxidative stress	MPO [40,41]	chest pain n = 604 ACS n = 1090	4^th ^quartile OR = 4.1 for MACE at 6 months HR = 2.11 for death/MI at 6 months
	sPLA2 [42]	ACS n = 446	HR = 3.08 for death/MI
	Lp(a) [43]	CAG patients n = 504	RR = 2.47 for the presence of obstructive CAD
Fibrous cap degradation	MMP2 [44]	ACS n = 33, SAP n = 17, control n = 17	serial elevation 2× normal value in ACS during 1 week
	MMP 9 [44]	ACS n = 33, SAP n = 17, control n = 17	serial elevation 2× normal value in ACS during 1 week
	PAPP-A [45,46]	ACS n = 37 SAP n = 19 control n = 13 ACS n = 547 Chest pain n = 644	Higher levels in ACS versus SAP/control OR = 2.44 for 30 day death/MI OR = 2.32 for 30 day death/MI
Coagulation or trombocyt aggregation	vWF [9,47,48]	STEMI n = 153 STEMI n = 86 314 STEMI	vWF release higher in cardiovascular death vWF release OR 6.1 for 14 day composite endpointOR = 2.0 for 30 day maortality/MI (highest quartile vs quartiles 1–3)
	[49]	ACS 544 chest pain patients n = 626	OR = 2.71 for death/MI at 6 months OR = 6.65
Ischemia	IMA [50]	ACS n = 256)	negative predictive value = 96%
	FFA [51]	scheduled PCI n = 22	Correct prediction of ECG/troponin findings post procedure
	Choline [52]	suspected ACS n = 327	HR = 6.05 for cardiac death/non-fatal arrest in troponin negative group
	GP-BB [53,54]	chest pain n = 48 chest pain n = 107	Better prediction than CK-MB
	NT-proBNP [55,56]	ACS n = 1791 FRISC I ACS n = 2019 FRISC II	OR = 3.7 for death and non-fatal MI after 30 days HR = 4.1 for mortality after 2 years in non-invasive group OR = 1.55 with every standard deviation rise

CRP = (high sensitivity) C-Reactive Protein; IL6 = interleukin6; IL1 = interleukin1; MCP1= monocyte chemoattractant protein-1; IL18 = interleukin18; IL10 = interleukin10; SAA = Serum amyloid A; ICAM = intercellular adhesion molecule-1; VCAM = Vascular cell adhesion molecule-1; PlGF = Placental Growth Factor; MPO = myeloperoxidase; sPLA2 = Circulating secretory phospholipase A2 activity; Lp(a) = Lp(a) lipoprotein; MMP2 = matrix metalloproteases-2; MMP9 = matrix metalloproteases-9; PAPP-A = Pregnancy-associated plasma protein A; vWF = von Willebrand factor; NT-proBNP = N-terminal pro-brain natriuretic peptide; FFA = free fatty acids; IMA = Ischemia modified albumin GP-BB = Glycogen phosphorylase isoenzym BB.

ACS = acute coronary syndrome; UAP = unstable angina pectoris; SAP = stable angina pectoris. CAG = coronary artery angiogram

OR = odds ratio; RR = relative risk; HR = hazard ratio

### Profile of biomarkers and radiological markers

Biomarkers in acute chest pain patients could be related to: 1) inflammation, 2) oxidative stress; 3) fibrous cap degradation; 4) coagulation or trombocyte aggregation; 5) ischemia. The experience and opinions of which is the best biomarker reflecting one of the mentioned components in coronary syndromes is continuously changing. After inclusion of about 500 patients, we will test the biomarkers that will be considered the most reliable marker for a wide range of the mentioned components. We will use biomarkers that can be measured after defrosting, and are proven to be reliable and quickly measurable. Because opinions and measurement techniques are constantly changing, we are awaiting future developments before deciding which biomarkers are of interest [25].

We hypothesize that in acute chest pain patients a combination of biomarkers evaluating the above mentioned components in ACS would accurately predict the existence of advanced systemic and coronary atherosclerosis, the presence of an ACS, and the prognosis of the patients in the QICS at least one-year follow-up will be assessed by visits at the out-patient clinic and by consultation of the municipal population registry. Extreme high biomarkers values due to acute non-cardiac inflammation should be excluded. We will test individual biomarkers and a profile of biomarkers head tot head against outcome, calcium score, presentational symptoms and traditional risk factors. We will also test individual biomarkers and profiles of biomarkers on top of calcium score, presentational symptoms and traditional risk factors.

### Study objectives

The primary objective is to evaluate whether a negative calcium score and/or a favourable biomarker profile can rule out the chance of a cardiac event in the following years. We hypothesize that EBT/MSCT and/or a biochemical profile can accurately rule out cardiac chest pain fast, (i.e. myocardial ischemia) and diagnose patients who will survive without myocardial infarctions in the following year. By ruling out myocardial ischemia it would prevent unnecessary hospital admittance and invasive or extensive ischemia testing, with all its costs and potential complications. Secondary objective is the identification of sensitive predictive markers in acute chest pain patients. We will test, presentational symptoms, traditional risk factors, individual biomarkers, a profile of biomarkers, coronary calcium score, coronary stenosis/plaque volume. Biomarkers will be retrospectively tested after defrosting of deep frozen blood taken on presentation.

## Methods/Design

**Figure 1:**
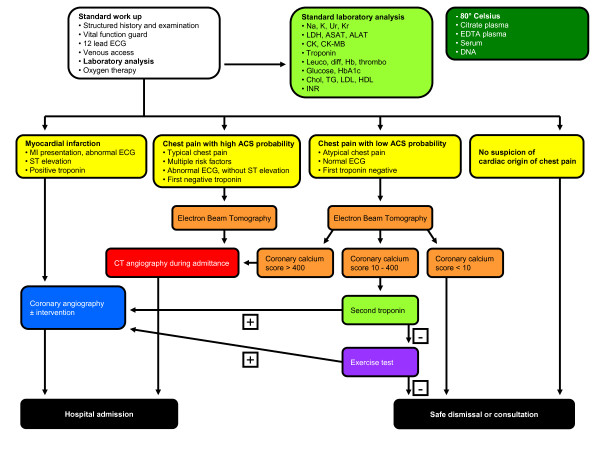
**Flow chart for chest pain patients referred to the emergency ward**. Na = sodium; K = potassium; Ur = ureum; KR = creatinin; LDH = lactatedehydrogenase, ASAT = Aspertate-aminotransferase, ALAT = Alanine-aminotransferase, CPK = Creatinekinase, CPK-MB = Creatinekinase -MB subunit, Leuco= leucocytes, Diff = blood smear differentiation, Hb = haemoglobin, thrombo = trombocytes Cho = total cholesterol, Tgl = triglycerides, LDC = low density cholesterol, HDC = high Density cholesterol, RHD = ratio of Cho/HDL, EDTA blood = ethylenediamine-tetraacetic acid blood tube.

All patients presenting with new onset acute chest pain in the emergency department will enter the flowchart as shown in figure 1. The flow chart has primarily been set-up to investigate cardiac causes of chest pain and to triage patients who need or need not undergo a cardiac catheterisation. Patients in whom a non-cardiac cause is suspected will of course be further evaluated. This is beyond the scope of this study protocol. Also patients admitted for ST elevation myocardial infarction will be included at the time of coronary angiography at primary percutaneous coronary intervention (PCI); these patients will serve as a positive control. A complete registration of each patient will be obtained. Baseline demographic and clinical characteristics will be collected, including age, gender, heart rate, systolic and diastolic blood pressure, weight, length, heart sounds and lung sounds, history of coronary artery bypass grafting (CABG), previous PCI, myocardial infarction and stroke, existence of all traditional risk factors, nature and duration of chest pain, electrocardiographic signs of ischemia, time of onset of symptoms and time of hospital admission. Additional investigation of ischemia detection in follow up will be recorded. The final diagnosis non cardiac chest pain, unstable angina, non ST elevation myocardial infarction, and ST elevation myocardial infarction, with registration of troponin and creatine kinase levels, lipid profiles, invasive diagnostic results, short term outcome, and long term outcome will also be recorded. For storage of frozen blood we requested ethical approval from the local ethical committee. The ethical committee waved ethical approval, because storage of blood is performed as standard for future additional testing, in order to evaluate future clinical important diagnostic considerations.

### In- and exclusion criteria

#### Inclusion criteria

- New onset acute chest pain without diagnostic ECG testing

- If there is a diagnostic ECG for ischemia, including ST elevation myocardial infarction, no further delay is warranted and patients will be treated accordingly without CT analysis. Registration of baseline characteristics, collection of blood to be frozen and follow up will be obtained.

#### Exclusion criteria are

- If there is diagnostic testing of a non cardiac cause of chest pain, or chest pain is evidently not cardiac, patients will be transferred to other specialists without cardiac CT testing.

- The existence of a life-threatening disease with a life expectancy of less than 6 months.

All chest pain patients without exclusion criteria and without cardiac history will undergo an EBT for coronary artery calcium assessment. A coronary calcium score > 10 is considered positive and will be evaluated by ischemia testing after ruling out infarction by troponin testing 6 hours after the start of chest pain. A coronary calcium score > 400 is considered to justify MSCT testing in order to rule out CAD. Depending on MSCT results and clinical findings during further hospital admittance coronary angiography will be performed.

The prognostic value of EBT calcium score will be evaluated during follow up. In case MSCT is performed, plaque characteristics will be evaluated and related to follow up. Biomarkers will be tested and related to follow up as well as to CT findings.

### EBT Protocol

Scans will be performed with an e-Speed EBT scanner (e-Speed, Imatron, San Francisco, CA, USA), in the dual 1.5 mm slice mode with an image acquisition time of 50 ms or 100 ms depending on patient size. EBT data will be collected during end-inspiratory breath hold. The non-enhanced scan will be ECG-triggered at 42% of the R-R interval. A set of 40 continuous axial coupes will be obtained starting directly under the trachea bifurcation until under the heart. Calcification will be defined as four adjacent pixels with density >130 Hounsfield units. Coronary artery calcification score will be calculated according to the method of Agatston using dedicated software.

### Multislice MDCT Protocol

All patients are imaged with a 64-slice MDCT scanner (Siemens) after premeditation with oral beta-blocker and/or intravenous metoprolol when needed. Thoracic CT angiography with retrospective gating will subsequently be performed with a single biphasic injection of 130 mL of iso-osmolar contrast material (100 mL at 5 mL/sec and 30 mL at 3 mL/sec) in caudal-to-cranial acquisition. Coronary, aortic, and pulmonary arterial attenuation values will be obtained. Coronary venous and right atrial enhancement will be evaluated to assess whether there is interference with coronary artery evaluation.

### Dual Source Computed Tomography: Procedure and Analysis

A retrospectively ECG-triggered DSCT scan (Somatom Definition, Siemens Medical Systems, Forchheim, Germany) with contrast enhancement will be made with a beam current of 340 mAs/rot, tube voltage of 120 kV, and a rotation time of 0.33 s for both tubes. ECG pulsing window will be 20–70% of the RR-interval for all patients. Scans will be made with a slice thickness of 3.0 mm with a 64 × 0.6 collimation. Scan direction will be was cranio-caudal starting above the coronary ostia and ending at the diaphragm below all cardiac structures. For the contrast enhanced scans 73 ml of non-ionic contrast agent (Iomeprol 400 mg I/ml, Iomeron^® ^400, Bracco, Italy) will be injected in an antecubital vein. Bolus triggering will be used for timing with the region of interest set in the descending aorta. A 50 ml saline bolus will follow the contrast bolus to maintain a compact bolus and to reduce the necessary amount of contrast. All patients will be treated with sublingual nitroglycerin (0.4 mg) just prior to the scan protocol.

Multi-phase reconstructions will be made to select the phase with least motion artefacts to obtain motion-free images of the coronary arteries. In this phase of the RR-interval final reconstructions will be made for evaluation. Images will be reconstructed using a B26f kernel, 0.6 slice thickness and 0.4 mm increment. 16 segments of the coronary artery will be evaluated according to the model of the American heart Association. All evaluable segments will be classified as normal (smooth borders), as having non-significant disease (luminal irregularities resulting in diameter narrowing < 50%), or as having significant stenosis (luminal diameter narrowing ≥ 50%).

### Study design

This is a single center prospective open trial.

### Number of patients

We will perform an interim analysis using after 100 and 500 patients.

### Duration of study

Patient recruitment has started in January 2006 and is expected to be completed in July 2009. Analysis and reporting is to be completed in November 2009.

### Participating center

The study will take place at the University Medical Center Groningen a referral center for primary PCI in STEMI patients, serving an area in the North of the Netherlands with approximately 1,2 million inhabitants.

## Discussion

A multimarker strategy in the evaluation of acute chest pain patients seems promising. [23] In addition, non-invasive imaging in the acute setting may prove additonal value. [26] Combination of these two approaches may further improve diagnostic accuracy and prevent unnecessary catheterisations and reduce the number of misdiagnosed infarctions.

The present study set-up may further elucidate the value of the proposed combination of biocemical and non-invasive diagnostics.

## Competing interests

None declared by the authors.

Financial competing interests: None declared. The study is not financially funded and not reimbursed by any other organization. Participation of the authors is completely voluntarily and ideological, in article writing as well as in additional radiological and biochemical testing. We will use the frozen blood specimens anonymously for biomarker testing.

## Authors' contributions

HMW: data collection, and analysis, writing the manuscript; principle investigator. GdJ: EBT and MSCT data collection and analysis, RAT: has made substantial contributions to concept and design of the study, has been involved in drafting the manuscript and revising it critically for important intellectual content; principle investigator, WN MD: practical implementation, has made substantial contributions to conception and design, and acquisition of data, IP: has been involved in drafting the manuscript and revising it critically for important intellectual content and has given intellectual input from biochemistry department, IvdH: intellectual input from cardiology department; has been involved in drafting the manuscript and revising it critically for important intellectual content, MO: has been involved in drafting the manuscript and revising it critically for important intellectual content and has given intellectual input from radiology department, FZ: has been involved in drafting the manuscript and revising it critically for important intellectual content. All authors have given final approval of the version to be published.

## Pre-publication history

The pre-publication history for this paper can be accessed here:



## References

[B1] Kamineni R, Alpert JS (2004). Acute coronary syndromes: initial evaluation and risk stratification. Prog Cardiovasc Dis.

[B2] Miller CD, Lindsell CJ, Khandelwal S, Chandra A, Pollack CV, Tiffany BR (2004). Is the initial diagnostic impression of "noncardiac chest pain" adequate to exclude cardiac disease?. Ann Emerg Med.

[B3] Shoyeb A, Bokhari S, Sullivan J, Hurley E, Miesner B, Pia R (2003). Value of definitive diagnostic testing in the evaluation of patients presenting to the emergency department with chest pain. Am J Cardiol.

[B4] O'Rourke RA, Brundage BH, Froelicher VF, Greenland P, Grundy SM, Hachamovitch R (2000). American College of Cardiology/American Heart Association Expert Consensus document on electron-beam computed tomography for the diagnosis and prognosis of coronary artery disease. Circulation.

[B5] Hunold P, Vogt FM, Schmermund A, Debatin JF, Kerkhoff G, Budde T (2003). Radiation exposure during cardiac CT: effective doses at multi-detector row CT and electron-beam CT. Radiology.

[B6] Mitka M (2004). Standards set for CT calcium screening but its clinical value remains unclear. JAMA.

[B7] Detrano RC, Wong ND, Doherty TM, Shavelle RM, Tang W, Ginzton LE (1999). Coronary calcium does not accurately predict near-term future coronary events in high-risk adults. Circulation.

[B8] Haberl R, Becker A, Leber A, Knez A, Becker C, Lang C (2001). Correlation of coronary calcification and angiographically documented stenoses in patients with suspected coronary artery disease: results of 1,764 patients. J Am Coll Cardiol.

[B9] Arad Y, Goodman KJ, Roth M, Newstein D, Guerci AD (2005). Coronary calcification, coronary disease risk factors, C-reactive protein, and atherosclerotic cardiovascular disease events: the St. Francis Heart Study. J Am Coll Cardiol.

[B10] Achenbach S (2006). Computed tomography coronary angiography. J Am Coll Cardiol.

[B11] Ghersin E, Litmanovich D, Dragu R, Rispler S, Lessick J, Ofer A (2006). 16-MDCT coronary angiography versus invasive coronary angiography in acute chest pain syndrome: a blinded prospective study. AJR Am J Roentgenol.

[B12] Hoffmann U, Nagurney JT, Moselewski F, Pena A, Ferencik M, Chae CU (2006). Coronary multidetector computed tomography in the assessment of patients with acute chest pain. Circulation.

[B13] Kuettner A, Beck T, Drosch T, Kettering K, Heuschmid M, Burgstahler C (2005). Diagnostic accuracy of noninvasive coronary imaging using 16-detector slice spiral computed tomography with 188 ms temporal resolution. J Am Coll Cardiol.

[B14] Rubinshtein R, Halon DA, Gaspar T, Jaffe R, Karkabi B, Flugelman MY (2007). Usefulness of 64-slice cardiac computed tomographic angiography for diagnosing acute coronary syndromes and predicting clinical outcome in emergency department patients with chest pain of uncertain origin. Circulation.

[B15] Gallagher MJ, Ross MA, Raff GL, Goldstein JA, O'Neill WW, O'Neil B (2007). The diagnostic accuracy of 64-slice computed tomography coronary angiography compared with stress nuclear imaging in emergency department low-risk chest pain patients. Ann Emerg Med.

[B16] Leber AW, Becker A, Knez A, von Ziegler F, Sirol M, Nikolaou K (2006). Accuracy of 64-slice computed tomography to classify and quantify plaque volumes in the proximal coronary system: a comparative study using intravascular ultrasound. J Am Coll Cardiol.

[B17] McLaughlin VV, Balogh T, Rich S (1999). Utility of electron beam computed tomography to stratify patients presenting to the emergency room with chest pain. Am J Cardiol.

[B18] Laudon DA, Vukov LF, Breen JF, Rumberger JA, Wollan PC, Sheedy PF (1999). Use of electron-beam computed tomography in the evaluation of chest pain patients in the emergency department. Ann Emerg Med.

[B19] Georgiou D, Budoff MJ, Kaufer E, Kennedy JM, Lu B, Brundage BH (2001). Screening patients with chest pain in the emergency department using electron beam tomography: a follow-up study. J Am Coll Cardiol.

[B20] Becker CR, Kleffel T, Crispin A, Knez A, Young J, Schoepf UJ (2001). Coronary artery calcium measurement: agreement of multirow detector and electron beam CT. AJR Am J Roentgenol.

[B21] Savino G, Herzog C, Costello P, Schoepf UJ (2006). 64 slice cardiovascular CT in the Emergency Department: concepts and first experiences. Radiol Med.

[B22] Jeudy J, White CS (2007). Evaluation of acute chest pain in the emergency department: utility of multidetector computed tomography. Semin Ultrasound CT MR.

[B23] Apple FS, Pearce LA, Chung A, Ler R, Murakami MM (2007). Multiple biomarker use for detection of adverse events in patients presenting with symptoms suggestive of acute coronary syndrome. Clin Chem.

[B24] Lindahl B, Toss H, Siegbahn A, Venge P, Wallentin L (2000). Markers of myocardial damage and inflammation in relation to long-term mortality in unstable coronary artery disease. FRISC Study Group. Fragmin during Instability in Coronary Artery Disease. N Engl J Med.

[B25] Jaffe AS, Babuin L, Apple FS (2006). Biomarkers in acute cardiac disease: the present and the future. J Am Coll Cardiol.

[B26] Dorgelo J, Willems TP, Geluk CA, van Ooijen PM, Zijlstra F, Oudkerk M (2005). Multidetector computed tomography-guided treatment strategy in patients with non-ST elevation acute coronary syndromes: a pilot study. Eur Radiol.

[B27] Pai JK, Pischon T, Ma J, Manson JE, Hankinson SE, Joshipura K (2004). Inflammatory markers and the risk of coronary heart disease in men and women. N Engl J Med.

[B28] Toss H, Lindahl B, Siegbahn A, Wallentin L (1997). Prognostic influence of increased fibrinogen and C-reactive protein levels in unstable coronary artery disease. FRISC Study Group. Fragmin during Instability in Coronary Artery Disease. Circulation.

[B29] Biasucci LM, Liuzzo G, Fantuzzi G, Caligiuri G, Rebuzzi AG, Ginnetti F (1999). Increasing levels of interleukin (IL)-1Ra and IL-6 during the first 2 days of hospitalization in unstable angina are associated with increased risk of in-hospital coronary events. Circulation.

[B30] Lindmark E, Diderholm E, Wallentin L, Siegbahn A (2001). Relationship between interleukin 6 and mortality in patients with unstable coronary artery disease: effects of an early invasive or noninvasive strategy. JAMA.

[B31] de Lemos JA, Morrow DA, Sabatine MS, Murphy SA, Gibson CM, Antman EM (2003). Association between plasma levels of monocyte chemoattractant protein-1 and long-term clinical outcomes in patients with acute coronary syndromes. Circulation.

[B32] Blankenberg S, Tiret L, Bickel C, Peetz D, Cambien F, Meyer J (2002). Interleukin-18 is a strong predictor of cardiovascular death in stable and unstable angina. Circulation.

[B33] Heeschen C, Dimmeler S, Hamm CW, Fichtlscherer S, Boersma E, Simoons ML (2003). Serum level of the antiinflammatory cytokine interleukin-10 is an important prognostic determinant in patients with acute coronary syndromes. Circulation.

[B34] Morrow DA, Rifai N, Antman EM, Weiner DL, McCabe CH, Cannon CP (2000). Serum amyloid A predicts early mortality in acute coronary syndromes: A TIMI 11A substudy. J Am Coll Cardiol.

[B35] Blankenberg S, Rupprecht HJ, Bickel C, Peetz D, Hafner G, Tiret L (2001). Circulating cell adhesion molecules and death in patients with coronary artery disease. Circulation.

[B36] Mulvihill NT, Foley JB, Murphy R, Crean P, Walsh M (2000). Evidence of prolonged inflammation in unstable angina and non-Q wave myocardial infarction. J Am Coll Cardiol.

[B37] Mulvihill NT, Foley JB, Murphy RT, Curtin R, Crean PA, Walsh M (2001). Risk stratification in unstable angina and non-Q wave myocardial infarction using soluble cell adhesion molecules. Heart.

[B38] Rallidis LS, Gika HI, Zolindaki MG, Xydas TA, Paravolidakis KE, Velissaridou AH (2003). Usefulness of elevated levels of soluble vascular cell adhesion molecule-1 in predicting in-hospital prognosis in patients with unstable angina pectoris. Am J Cardiol.

[B39] Lenderink T, Heeschen C, Fichtlscherer S, Dimmeler S, Hamm CW, Zeiher AM (2006). Elevated placental growth factor levels are associated with adverse outcomes at four-year follow-up in patients with acute coronary syndromes. J Am Coll Cardiol.

[B40] Baldus S, Heeschen C, Meinertz T, Zeiher AM, Eiserich JP, Munzel T (2003). Myeloperoxidase serum levels predict risk in patients with acute coronary syndromes. Circulation.

[B41] Brennan ML, Penn MS, Van Lente F, Nambi V, Shishehbor MH, Aviles RJ (2003). Prognostic value of myeloperoxidase in patients with chest pain. N Engl J Med.

[B42] Mallat Z, Steg PG, Benessiano J, Tanguy ML, Fox KA, Collet JP (2005). Circulating secretory phospholipase A2 activity predicts recurrent events in patients with severe acute coronary syndromes. J Am Coll Cardiol.

[B43] Tsimikas S, Brilakis ES, Miller ER, McConnell JP, Lennon RJ, Kornman KS (2005). Oxidized phospholipids, Lp(a) lipoprotein, and coronary artery disease. N Engl J Med.

[B44] Kai H, Ikeda H, Yasukawa H, Kai M, Seki Y, Kuwahara F (1998). Peripheral blood levels of matrix metalloproteases-2 and -9 are elevated in patients with acute coronary syndromes. J Am Coll Cardiol.

[B45] Bayes-Genis A, Conover CA, Overgaard MT, Bailey KR, Christiansen M, Holmes DR (2001). Pregnancy-associated plasma protein A as a marker of acute coronary syndromes. N Engl J Med.

[B46] Heeschen C, Dimmeler S, Hamm CW, Fichtlscherer S, Simoons ML, Zeiher AM (2005). Pregnancy-associated plasma protein-A levels in patients with acute coronary syndromes: comparison with markers of systemic inflammation, platelet activation, and myocardial necrosis. J Am Coll Cardiol.

[B47] Collet JP, Montalescot G, Vicaut E, Ankri A, Walylo F, Lesty C (2003). Acute release of plasminogen activator inhibitor-1 in ST-segment elevation myocardial infarction predicts mortality. Circulation.

[B48] Montalescot G, Philippe F, Ankri A, Vicaut E, Bearez E, Poulard JE (1998). Early increase of von Willebrand factor predicts adverse outcome in unstable coronary artery disease: beneficial effects of enoxaparin. French Investigators of the ESSENCE Trial. Circulation.

[B49] Heeschen C, Dimmeler S, Hamm CW, Brand MJ van den, Boersma E, Zeiher AM (2003). Soluble CD40 ligand in acute coronary syndromes. N Engl J Med.

[B50] Christenson RH, Duh SH, Sanhai WR, Wu AH, Holtman V, Painter P (2001). Characteristics of an Albumin Cobalt Binding Test for assessment of acute coronary syndrome patients: a multicenter study. Clin Chem.

[B51] Kleinfeld AM, Prothro D, Brown DL, Davis RC, Richieri GV, DeMaria A (1996). Increases in serum unbound free fatty acid levels following coronary angioplasty. Am J Cardiol.

[B52] Danne O, Mockel M, Lueders C, Mugge C, Zschunke GA, Lufft H (2003). Prognostic implications of elevated whole blood choline levels in acute coronary syndromes. Am J Cardiol.

[B53] Mair J, Puschendorf B, Smidt J, Lechleitner P, Dienstl F, Noll F (1994). Early release of glycogen phosphorylase in patients with unstable angina and transient ST-T alterations. Br Heart J.

[B54] Rabitzsch G, Mair J, Lechleitner P, Noll F, Hofmann U, Krause EG (1995). Immunoenzymometric assay of human glycogen phosphorylase isoenzyme BB in diagnosis of ischemic myocardial injury. Clin Chem.

[B55] Heeschen C, Hamm CW, Mitrovic V, Lantelme NH, White HD (2004). N-terminal pro-B-type natriuretic peptide levels for dynamic risk stratification of patients with acute coronary syndromes. Circulation.

[B56] Jernberg T, Lindahl B, Siegbahn A, Andren B, Frostfeldt G, Lagerqvist B (2003). N-terminal pro-brain natriuretic peptide in relation to inflammation, myocardial necrosis, and the effect of an invasive strategy in unstable coronary artery disease. J Am Coll Cardiol.

